# Euglycemic Diabetic Ketoacidosis in a 27 year-old female patient with type-1-Diabetes treated with sodium-glucose cotransporter-2 (SGLT2) inhibitor Canagliflozin

**DOI:** 10.12669/pjms.323.9201

**Published:** 2016

**Authors:** Nimrah Bader, Lubna Mirza

**Affiliations:** 1 Nimrah Bader, Medical Student. Aga Khan University Hospital, Karachi, Pakistan; 2 Lubna Mirza, MD. Norman Regional Hospital, Norman, Oklahoma, USA

**Keywords:** Diabetes, Diabetic Ketoacidosis (DKA), SGLT-2 Inhibitors

## Abstract

We are reporting a timely case of atypical euglycemic diabetic ketoacidosis in a type 1 diabetic patient treated with sodium-glucose cotransporter-2 (SGLT-2) inhibitor canagliflozin. The clinical history, physical examination findings and laboratory values are described. Other causes of acidosis such as salicylate toxicity or alcohol intoxication were excluded. Ketoacidosis resolved after increasing dextrose and insulin doses supporting the hypothesis that SGLT-2 inhibitors may lead to hypoinsulinemia. Euglycemic ketoacidosis did not recur in our patient after discontinuing canagliflozin. We recommend reserving SGLT2 inhibitor therapy to type 2 diabetics, discontinuing medication and treating patients presenting with ketoacidosis due to SGLT-2 inhibitors with higher concentrations of dextrose with appropriate doses of insulin to help resolve acidosis.

## INTRODUCTION

Sodium glucose co-transporter 2 (SGLT2) inhibitors are one of the newest anti-diabetic drugs that improve glycemic control by increasing urinary excretion of glucose. SGLT2 inhibitors are indicated for type 2 diabetics as adjunct to diet and exercise. They improve weight and blood pressure as well with glucosuria. Since the launch of the medication, several cases of diabetes ketoacidosis (DKA) have emerged. We present a case of type 1 diabetic patient treated with SGLT2 inhibitor who was admitted to the hospital with DKA. Literature review shows since the approval of drug class in 2013, these drugs increase risk for DKA. The risk is higher in autoimmune diabetes. In May 2015, the Food and Drug Administration issued a warning that SGLT2 inhibitors may lead to ketoacidosis.

## METHOD

A literature search was conducted on the Clin-Alert database for adverse reactions and PubMed using keywords ‘SGLT2’; ‘canagliflozin’ and ‘ketoacidosis’. Results revealed two cases where ketoacidosis was seen in Type 2 diabetics after they were started on an SGLT-2 Inhibtor.^1^ Possible mechanism behind the euglycemic ketoacidosis was also identified.^2^ We hope that writing this case report may highlight this potential side effect in Type 1 Diabetics as well. Our case report features a young woman with type 1 diabetes who developed ketoacidosis eight months after starting canagliflozin.

## CASE REPORT

A 27-year-old Caucasian woman with type 1 diabetes was admitted to the hospital with ketoacidosis. She was first diagnosed with type 1-diabetes 15 years ago. She was using an insulin pump since age 18. Past medical history was significant for primary hypothyroidism and depression. She was started on sodium-glucose cotransporter-2 (SGLT2) inhibitor canaglifozin 8 months earlier by Endocrinologist. After starting treatment with canaglifozin her insulin requirement went down. She lost about four pounds of weight and her hemoglobin A1C improved slightly from 8.6% to 8.4% six months after starting canagliflozin. The day before presentation she worked the night shift, slept all day the next day and woke up at 5:00 p.m. She felt nauseated with heavy breathing and muscular pains. She also noticed spots in her vision. Her blood sugar was normal at 114 mg/dl. She had eaten a grilled cheese sandwich before going to sleep along with two beers. No history of aspirin use. She worked as a nurse and drank alcohol only three to four times a year. There was a positive family history for autoimmune hypothyroidism in her mother. She had no known drug allergies and her home medications included Dexvenlafaxine 50mg orally for depression at bedtime and Levothyroxine 150mcg orally once daily for hypothyroidism.

She was treated with intravenous 5% Dextrose with half normal saline and insulin in the emergency room before being transferred to the Intensive care unit, but the anion gap didn’t close despite continued intravenous 5% Dextrose and insulin administration 24 hours later. At this point Endocrinology services were consulted. She was found to be in overall stable condition. Her Temperature was 36.8 degrees centigrade, pulse was 72 beats per minute, respiratory rate was 16, Blood pressure 131/73 and pulse ox was 98% on room air. She was awake and in no distress. Physical examination was unremarkable.

### Laboratories Studies

Sodium 134, potassium 3.9, chloride 110, CO2 12, glucose 132mg/dl, calcium 8.2mg/dl, TSH 0.92 with free T4 of 0.99. Anion gap was 16, which was improved from 20 on admission, but had not changed in the last 24 hours. Beta hydroxybutyrate were positive. Corticotrophin stimulation test ruled out adrenal insufficiency and serum salicylate levels were not high. Toxicology screen was negative for alcohol or other commonly abused illicit drugs.

Canagliflozin use was suspected as a likely reason for development of resistant and unusual diabetes ketoacidosis episode in this patient. High fat meal and use of alcohol were linked to triggering of this episode. We recommended increasing dextrose infusion to 10% to help expedite removal of beta hydroxybutyrate. By next morning her anion gap closed, she felt better and she was discharged home. She stayed off of SGLT2 inhibitor and didn’t have any further episodes of euglycemic ketoacidosis.

## DISCUSSION

Sodium glucose transporters -2 (SGLT-2) inhibitors have recently emerged as a novel way to treat diabetes and improve glycemic control. Canagliflozin was the first drug in this class approved by the Food and Drug Administration (FDA) in March 2013.^3^ SGLTs are a family of sodium glucose co-transporters. SGLT-2 is present almost exclusively in brush border of epithelial cells in the proximal tubule of the kidney. It is responsible for reabsorbing around 90% of filtered glucose. SGLT-2 inhibitors exploit this phenomenon by antagonizing the transporter.^4^ This leads to increased glucose excretion, improving glycemic control.^5^ While the use of SGLT-2 inhibitors in Type 2 diabetics has been extensively studied both as a single agent and in combination in terms of efficacy and safety,^6^-^10^ several independent smaller studies have shown benefit in Type 1 Diabetics as well.^11^,^12^ Our clinical experience in treating a patient with Type 1 diabetes resulted in an unexpected side effect – euglycemic diabetic ketoacidosis.

Diabetic ketoacidosis (DKA) is a life threatening condition characterized by uncontrolled hyperglycemia, increased levels of ketones and metabolic acidosis. DKA is much less commonly seen in type 2 diabetes as compared to type 1 diabetics. The mechanism behind DKA is hormonal derangements between insulin and other counter regulatory hormones like glucagon, cortisol, growth hormone and catecholamines.^13^ In the case of our type 1 diabetic patient, glucose levels were normal suggesting this was an atypical case of DKA.

SGLT-2 inhibitors help improve glycemic control in diabetic patients by preventing renal glucose re-absorption as mentioned earlier in the paper. However, treatment in subjects with type 2 diabetes increases both plasma glucagon and endogenous glucose production, which may be the cause behind the normoglycemia during atypical DKA. Biological research on this factor has mostly focused on islet cells of Type 2 diabetics.

The exact mechanisms are unknown at present time. In a recently published study in Nature, it is demonstrated that SGLT2 is expressed in glucagon-secreting alpha cells of the pancreatic islets.^14^ The investigators further found that expression of SLC5A2 (which encodes SGLT2) was lower and glucagon (GCG) gene expression was higher in islets from type 2 diabetic individuals and in normal islets exposed to chronic hyperglycemia than in islets from non-diabetics. In addition SGLT-2 inhibitor treatment in human islets triggered glucagon secretion through KATP channel activation. The investigators also found that dapagliflozin treatment further promotes glucagon secretion and hepatic gluconeogenesis in healthy mice, thereby limiting the decrease of plasma glucose induced by fasting. This may be a possible mechanism behind the masking of hyperglycemia in this patient.

## CONCLUSIONS

SGLT2 inhibitors are an exciting new drug class that is safe and efficient in most type 2 diabetes patients. SGLT2 inhibitors can cause DKA in both type 1 and type 2 diabetes. Patients and healthcare providers need to be educated on signs and symptoms of DKA associated with SGLT2 inhibitors for reducing patient morbidity and mortality.
